# Intragroup competition predicts individual foraging specialisation in a group‐living mammal

**DOI:** 10.1111/ele.12933

**Published:** 2018-03-14

**Authors:** Catherine E. Sheppard, Richard Inger, Robbie A. McDonald, Sam Barker, Andrew L. Jackson, Faye J. Thompson, Emma I. K. Vitikainen, Michael A. Cant, Harry H. Marshall

**Affiliations:** ^1^ Centre for Ecology and Conservation University of Exeter Penryn Campus Cornwall TR10 9FE UK; ^2^ Environment and Sustainability Institute University of Exeter Penryn Campus Cornwall TR10 9FE UK; ^3^ Department of Zoology School of Natural Sciences Trinity College Dublin Dublin 2 Ireland; ^4^ Department of Biosciences University of Helsinki PO Box 65 (Viikinkaari 1) Helsinki FI‐00014 Finland; ^5^ Centre for Research in Ecology, Evolution and Behaviour University of Roehampton London SW15 4JD UK

**Keywords:** Banded mongoose, competition, foraging behaviour, foraging niche, group‐living, *Mungos mungo*, social group, specialisation, stable isotope

## Abstract

Individual foraging specialisation has important ecological implications, but its causes in group‐living species are unclear. One of the major consequences of group living is increased intragroup competition for resources. Foraging theory predicts that with increased competition, individuals should add new prey items to their diet, widening their foraging niche (‘optimal foraging hypothesis’). However, classic competition theory suggests the opposite: that increased competition leads to niche partitioning and greater individual foraging specialisation (‘niche partitioning hypothesis’). We tested these opposing predictions in wild, group‐living banded mongooses (*Mungos mungo*), using stable isotope analysis of banded mongoose whiskers to quantify individual and group foraging niche. Individual foraging niche size declined with increasing group size, despite all groups having a similar overall niche size. Our findings support the prediction that competition promotes niche partitioning within social groups and suggest that individual foraging specialisation may play an important role in the formation of stable social groupings.

## Introduction

Within animal populations there is often remarkable heterogeneity in foraging behaviour (birds: Harris *et al*. 2014; sharks: Matich *et al*. 2011; mammals: Newsome *et al*. 2009; Robertson *et al*. 2014, 2015). This intraspecific variation in foraging niche can often be attributed to differences in sex (Bearhop *et al*. 2006; Stauss *et al*. 2012), age (Newland *et al*. 2009) or morphology (Pegg *et al*. 2015). However, where an individual's niche is substantially narrower than the population's for reasons not attributed to age, sex or morphology, it is termed individual specialisation (Bolnick *et al*. 2003).

Individual specialisation in foraging niche has important implications for ecology and evolution. Increased foraging specialisation is associated with both positive and negative effects such as improved reproductive success (Otterbeck *et al*. 2015; Pagani‐Núñez *et al*. 2015) and higher predation risk (Darimont *et al*. 2007). Individual foraging specialisation in European badgers *Meles meles* has been found to improve body condition when competition is more intense (Robertson *et al*. 2015), demonstrating how foraging specialisation can be beneficial and that between‐individual variation in foraging behaviour can have important individual‐level effects. However, while there has been considerable discussion on the causes and consequences of individual foraging specialisation in non‐social species (Bolnick *et al*. 2003; Araújo *et al*. 2011), the causes and consequences of such specialisation in species living in stable social groups are relatively poorly understood. Foraging specialisation in these species merits particular consideration as social group characteristics are likely to have a greater impact on individual ecological and evolutionary processes, such as individual specialisation, than the characteristics of the whole population, made up of multiple social groups (Chepko‐Sade & Halpin 1987; Johnstone & Cant 2010).

One of the major consequences of group living is increased proximity to, and interactions with, conspecifics, and so greater intragroup competition for resources (Krause & Ruxton 2002). Increased competition for resources is expected to have important effects on individual foraging specialisation (Svanbäck & Bolnick 2005; Svanbäck & Persson 2009; Araújo *et al*. 2011; Parent *et al*. 2014). Classic optimal foraging theory predicts that in the face of increased competition, individuals should add new prey items to their diet, widening their trophic niche and forming a population of generalist foragers (Stephens & Krebs 1986). However, classic competition theory (e.g. niche partitioning: Schoener 1974; Pianka 1981) predicts that as competition increases, stable coexistence is achieved through niche differentiation, reducing dietary overlap between competitors (e.g. Svanbäck & Bolnick 2007). There is empirical support for both hypotheses from studies in non‐social species (Svanbäck & Bolnick 2007; Araújo *et al*. 2008, 2011; Tinker *et al*. 2008). Here, we use these hypotheses to form two opposing predictions about the effect of competition within social groups on individual foraging specialisation:

1. intragroup competition promotes generalist foraging behaviours and a reduction in individual foraging specialisation (hereafter termed the ‘optimal foraging hypothesis’);
2. intragroup competition leads to niche differentiation between conspecifics, increasing individual foraging specialisation (hereafter termed the ‘niche partitioning hypothesis’).

We test these hypotheses in a population of wild banded mongooses *Mungos mungo* by exploring the effects of group size, a proxy of intragroup competition, on individual isotopic niche size; a metric of foraging niche size (Bearhop *et al*. 2004). Banded mongooses live in mixed‐sex groups of typically 10–30 individual members occupying distinct territories (Cant *et al*. 2013, 2016). Our use of group size as a proxy for intragroup competition is supported by previous studies showing that individuals in larger groups have lower per capita reproductive success (Cant *et al*. 2010) and are at greater risk of aggressive eviction from the group (Thompson *et al*. 2016). We further test this assumption by investigating the effect of group size on individual weight. Banded mongooses present a good model species for testing how intragroup competition influences the development of individual specialisation in a group‐living species. They forage in close proximity to one another (10–20 m) and have a broad diet of invertebrates such as millipedes, ants and beetles, and occasionally vertebrates including frogs and reptiles (Rood 1975; Marshall *et al*. 2017). Previous studies on the system have also demonstrated the coexistence of multiple foraging strategies within banded mongoose groups (Müller & Cant 2010), suggestive of between‐individual variation in foraging niche.

## Materials and Methods

### Study system and sample collection

Our study was carried out on a population of wild banded mongooses on the Mweya Peninsula, Queen Elizabeth National Park, Uganda (0°12′ S, 29°54′ E). As part of a long‐term research project, life history data have been collected on this population since 1995. Below, we provide details specific to our study; for further information about banded mongoose biology and the study site, see Cant *et al*. (2013, 2016).

All mongooses are individually identified using unique hair‐shave patterns on their back and pit tags (TAG‐P‐122IJ, Wyre Micro Design Ltd., UK) inserted under the skin in the scruff of the neck. Each social group is visited at least every 3 days to collect basic life history data and groups containing heavily pregnant females are visited daily to record accurate birth dates. Most individuals are trained to step onto portable electronic scales in return for a small milk reward. Since June 2000 they have been weighed weekly on two consecutive mornings before they started foraging.

Between September 2013 and October 2015, 760 vibrissa samples were collected from 322 banded mongooses in 10 social groups. Individual mongooses were trapped using Tomahawk live traps (Tomahawk Live Trap Co., Tomahawk, Wisconsin, USA) and vibrissa samples were hand plucked under anaesthetic (isoflurane, Abbot Laboratories) as part of routine trapping (see Jordan *et al*. 2010 for details of trapping procedure). Banded mongoose vibrissae are small and so 4–5 vibrissae were collected in each sample to obtain enough material for stable isotope analysis (around 0.7 mg, see below). Vibrissae were sampled from the same side of each mongoose's snout to ensure fresh vibrissa growth was sampled at each trapping.

### Sample preparation and stable isotope analysis

We used stable isotope analysis to investigate patterns of individual isotopic niche within and between mongoose groups (Bearhop *et al*. 2003, 2004; Araújo *et al*. 2007; Newsome *et al*. 2007). Previous studies demonstrate that analysis of ^13^C and ^15^N stable isotopes provides an efficient method for measuring individual‐ and population‐level dietary niche (Newsome *et al*. 2009, 2015; Robertson *et al*. 2014). Isotopes of ^13^C and ^15^N vary with habitat and trophic level, respectively, representing foraging location and trophic position (Crawford *et al*. 2008). Repeated measurements of individual isotope values over time have been suggested as an indication of the degree of individual foraging specialisation (Bearhop *et al*. 2004). We repeatedly sampled banded mongoose vibrissae at each live trapping (mean ± SD resampling rate = 4.7 ± 2.8 months, *n* = 64 individuals). We established the rate of mongoose vibrissa growth by feeding six individuals with Rhodamine B‐infused carnivore kibble and collecting their vibrissae a month later. Rhodamine B is a commonly used biomarker which, once ingested, is incorporated in keratinous tissues as a fluorescent band visible using fluorescent microscopy (Fisher 1999; Robertson *et al*. 2013). This showed that mongoose whiskers have a mean regrowth time of 6.3 months (lower‐upper SE = 5.3–7.8 months). Consequently, vibrissae were approximately fully grown each time they were sampled. Each sample contained 4–5 vibrissae from an individual banded mongoose to provide enough material for the isotopic analysis. Vibrissae from each sample were scraped to remove debris and cut into small fragments using a scalpel and forceps. These small fragments were then mixed and weighed to around 0.7 mg (mean ± SD: 0.77 mg ± 0.33; *n* = 760) and sealed in small tin capsules (Elemental Microanalysis) for stable isotope analysis. Carbon and nitrogen isotope ratios (δ^13^C/δ^15^N) were determined using continuous flow isotope ratio monitoring mass spectrometry (CF‐IRMS), using a Sercon Integra integrated elemental analyser and mass spectrometer (Cheshire, UK). Isotope ratios are expressed as δ values, reporting parts per mil (‰), according to the equation

$$\delta X=(\left(R_{\mathrm{sample}}/R_{\mathrm{standard}}\right)-1)\times 1000$$

where X represents ^13^C or ^15^N, and R represents the ratio of heavy to light isotopes (^13^C/^12^C or ^15^N/^14^N). International reference materials (IAEA, Vienna) were analysed within each run for calibrating δ^13^C and δ^15^N sample values scaled to V‐PDB and air respectively.

### Statistical analysis

We tested our assumption that group size is a proxy for intragroup foraging competition in banded mongooses by fitting linear mixed effects models (LMM) predicting its effect on individual daily weight change and overall weight. We calculated daily weight change as the difference in an individual's weight between the two consecutive weekly morning weights. We used the first morning weight record as our overall weight measure. Group size was included as a fixed effect and measured as the number of individuals in the group on the first morning. The models also included the following fixed effects: individual age and sex to control for age and sex differences, and rainfall in the previous 60 days to control for differences in ecological conditions (Marshall *et al*. 2017). The weight change model also included weight on the first morning as a fixed effect to control for regression to the mean. Individual and group ID were included as random effects to control for repeated measures. We fitted these models to 12,592 weight records from 264 individuals in 11 groups measured between 2000 and 2016. The model residuals were normally distributed with homogenous variance.

We quantified dietary variability in individual mongooses using the 95% prediction ellipse area (ell95), representing the area of isotopic niche space occupied by the individual (Jackson *et al*. 2011). We calculated ell95c values (ell95 values corrected for sample size, Jackson *et al*. 2011) for all individual banded mongooses with four or more isotope samples (315 samples from 64 individuals; mean ± SD samples per individual = 4.92 ± 1.02, maximum = 7).

**Figure 1:**
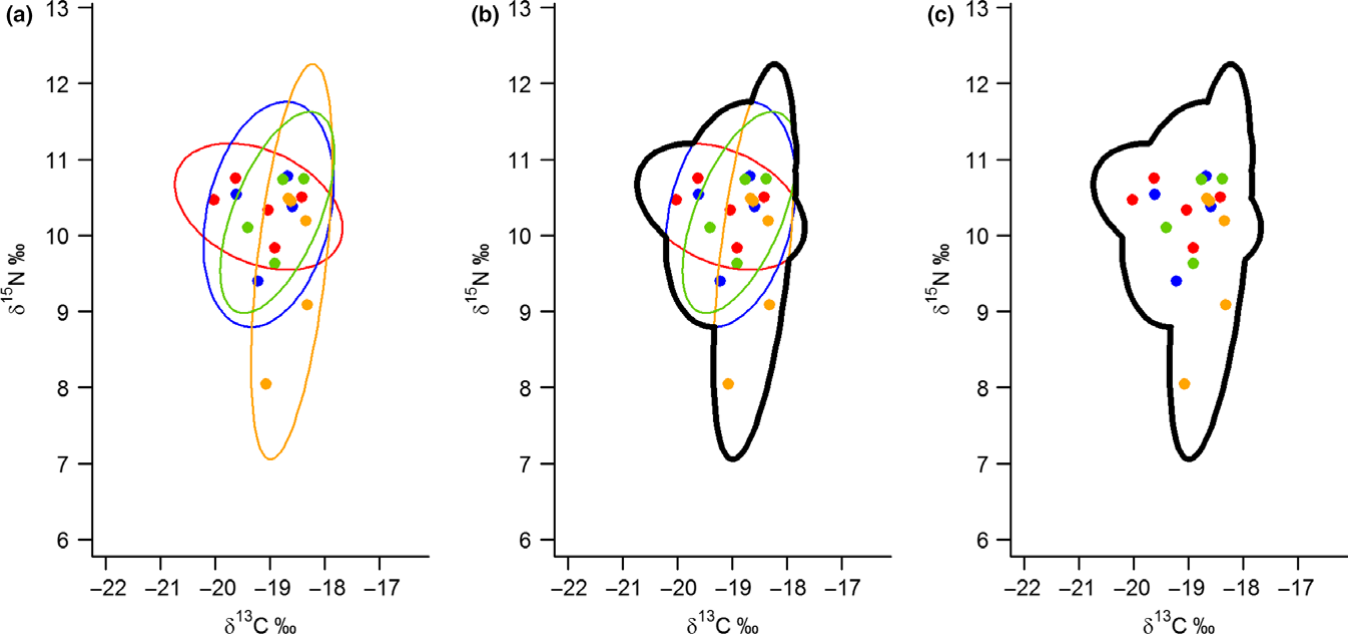
Calculating the relative individual niche index (RINI) in banded mongoose groups (*Mungos mungo*). (a) In each group (group 21 in this example) individual 95% prediction ellipse areas, corrected for sample size (ell95c, Jackson *et al*. 2011) were overlaid. The outline of the area these overlaid ellipses covered was calculated (b) to create a group niche area (c) which the individual ellipse areas in (a) were expressed as a proportion of. In each plot: colours represents different individuals; points represents carbon (δ^13^C) and nitrogen (δ^15^N) isotope ratios obtained from vibrissa samples (in this example 17 samples from four individuals); thin coloured lines show individual's 95% prediction ellipses; the think black line shows the estimated group niche area.

When investigating between‐individual variation in foraging niche size, it is important to consider individual niche size relative to the total niche the population occupies (Roughgarden 1972). In group‐living species, particularly species such as banded mongooses that forage as a group and are territorial (Thompson *et al*. 2017), it is more appropriate to consider individual niche relative to social group niche size rather than the broader population niche. We therefore calculated a relative individual niche index (RINI) by expressing each individual's ell95c as a proportion of the total area covered when all group members’ ell95c values were overlaid (Fig. 1). The total area is simply the shape defined by the curve derived from the union of all the group members ellipses which we calculated using the function union.owin from the R package spatstat (Baddeley *et al*. 2015). A helper function siberKapow and explanatory vignettes have been added to R package SIBER (Jackson & Parnell 2017) to perform these calculations, and produce figures visualising the process (Fig. 1). We used this area as the measure of group niche here rather than a separate ellipses calculated from the group's pooled isotope data, as it is then possible for one individual to have a larger ellipse than the entire group, and hence then an RINI value greater than 1 (i.e. suggesting individual niches larger than the group's niche).

Under our two competing hypotheses, increases in intragroup competition would be expected to lead to an increase (‘optimal foraging hypotheses’) or decrease (‘niche partitioning hypothesis’) in RINI. To test these predictions, we fitted a linear mixed effects models (LMMs) predicting RINI (square‐root transformed to meet the assumptions of normality) as a function of group size. Group size was calculated as the mean daily number of individuals in the social group across the range of dates vibrissae were sampled from each group (mean ± SD = 663 ± 101 days across nine social groups). Visual inspection showed that the distribution of these group size values was bimodal with the data clearly grouping into two sets above and below a group size of 17 (see Fig. 3b). Therefore, in addition to fitting group size as a continuous variable, we also fitted our model with group size categorised as ‘small’ (< 17 members, three groups) and ‘large’ (> 17 members, six groups) to ensure any group size effect in our continuous model were not the result of this bimodal distribution. Age (in years) and sex were also included as explanatory variables in the models to confirm that the variation in isotopic niche between individual mongooses was not age or sex specific, and therefore due to individual specialisation (Bolnick *et al*. 2003). The proportion of individuals from whom we were able to obtain a RINI value (those with four or more vibrissae) varied between social groups (mean = 34%, range = 13%–70%, *n* = 9 groups). We also included this proportion in our models to control for differences in group representation potentially influencing the estimate of group niche that each RINI value was expressed relative to. The proportion of individuals sampled in each groups was independent of overall group size (Spearman's ρ = − 0.033, *P* = 0.95, *n* = 9 groups; ‘small’ groups: mean = 33%, range = 21%–40%, *n* = 3 groups; ‘large’ groups: mean = 34%, range = 13%–70%, *n* = 6 groups). Environmental variation at Mweya is driven by rainfall which mainly falls in two wet season (March–May and August–December; Marshall *et al*. 2016). As individual ell95c values covered time periods encompassing a range of seasons (mean = 543 ± 132 days, range = 237–710 days), we did not need to account for seasonality in our models. Social group was included as a random effect to control for repeated measures and model residuals were normally distributed with homogenous variance.

To investigate the effect of competition on diet at the group level, we also fitted a linear model predicting group niche size as a function of number of group members. Group niche size was measured by calculating an ell95c for each group using all samples collected from each social group (760 samples from 322 individuals from 10 social groups). Number of group members was measured as the mean daily number of individuals in the group across the range of days the group was sampled. We also included this sampling time period in the model to control for any effects on group niche size due to one group being sampled over a longer time period than another (mean ± SD = 650 ± 104 days, *n* = 10 groups). As in the individual‐level analysis above, we also ran this analysis with groups categorised as ‘small’ (< 17 members) or ‘large’ (> 17). Both model residuals were normally distributed with homogenous variance.

The significance of each term in the models was assessed using likelihood ratio tests (mixed models) or F‐tests (non‐mixed models), comparing the full model to a model without a particular variable (Forstmeier & Schielzeth 2011). We did not reduce our models due to issues with stepwise model reduction (Whittingham *et al*. 2006; Mundry & Nunn 2009; Forstmeier & Schielzeth 2011). All analyses were undertaken in R version 3.4.1 (R Core Team 2017). We used the lme4 package version 1.1‐13 to fit LMMs (Bates *et al*. 2015) and calculated the r‐squared value for each mixed model (Nakagawa & Schielzeth 2013) using the MuMIn package (Barton 2016). We used the SIBER package version 2.1.3 to fit bivariate ellipses and calculate ell95c values (Jackson *et al*. 2011).

## Results

**Figure 2:**
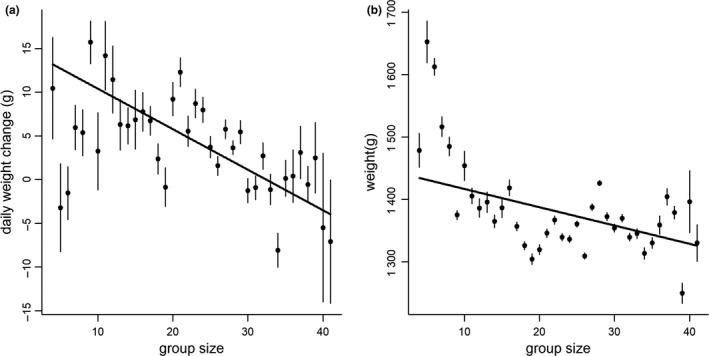
Individual banded mongooses (*Mungos mungo*) in larger groups (a) gained less weight day‐to‐day and (b) overall were in poorer condition. Points and error bars are the mean and standard errors (*n* = 12 592 weight records from 264 individuals in 11 groups measured between 2000 and 2016) for each group size and lines are the relationships predicted by our models with all other variables set at their mean.

Banded mongooses in larger groups gained less weight day‐to‐day (LMM: β ± SE = − 0.46 ± 0.054, χ^2^_1_ = 70.47, *P* < 0.001; Fig. 2a) and overall were in poorer condition (β ± SE = − 2.92 ± 0.19, χ^2^_1_ = 227.57, *P* < 0.001; Table 1; Fig. 2b). This supported our assumption that group size was a good proxy for intragroup foraging competition. Daily weight gains and overall weights were greater in older individuals and when there had been more rainfall (Table 1). Sex did not affect daily weight gains but overall males were heavier than females (Table 1). As expected by regression to the mean, weight gains were lower in individuals who were heavier at the first morning weighing (Table 1).

**Table 1:** Linear mixed effect models predicting individual daily weight change and overall weight in banded mongooses (conditional *r* ^2^ = 0.05 and 0.80)

Response	Effect	Estimate	SE	χ^2^	*P*
Daily weight change (g)	Intercept	58.959	3.70		
	**Age**	0.0035	0.0005	43.53	**4.17 × 10** ^−**11**^
	Sex (male)	−0.86	0.93	0.88	0.35
	**Weight on first morning**	−0.037	0.003	190.91	**<2.2 × 10** ^−**16**^
	**Rainfall in last 60 days**	0.033	0.005	47.20	**6.40 × 10** ^−**12**^
	**Group Size**	−0.46	0.054	70.47	**<2.2 × 10** ^−**16**^
Weight (g)	**Age**	0.058	0.002	1018.11	**<2.2 × 10** ^−**16**^
	**Sex (male)**	30.76	15.05	6.01	**0.014**
	**Rainfall in last 60 days**	0.36	0.013	713.38	**<2.2 × 10** ^−**16**^
	**Group Size**	−2.93	0.19	227.57	**<2.2 × 10** ^−**16**^

Significant effects are shown in bold.

**Figure 3:**
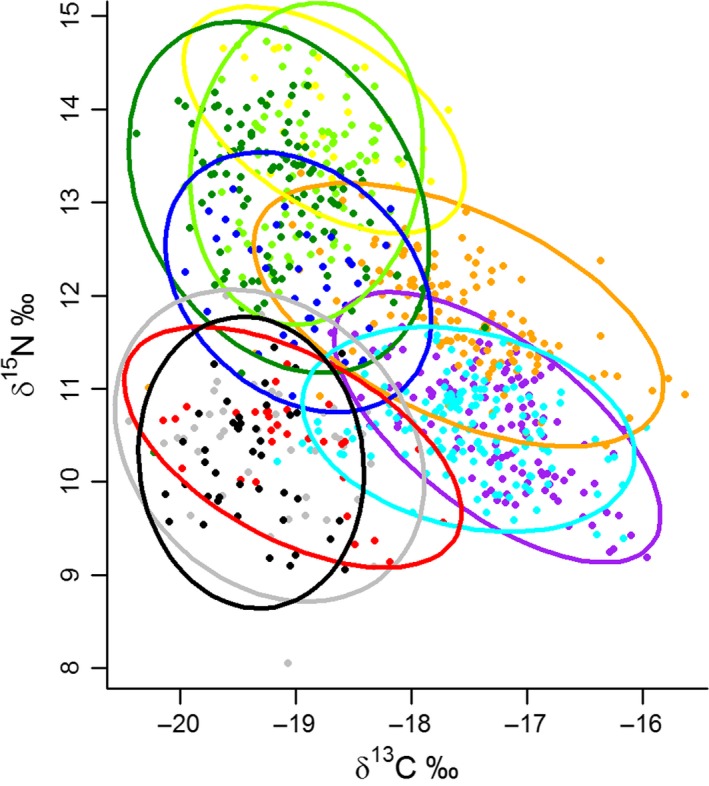
Banded mongoose (*Mungos mungo*) vibrissa nitrogen (δ^15^N) and carbon (δ^13^C) isotope ratios. The data are divided into social groups by colour. Each point represents one vibrissa sample collected from an individual (760 vibrissa samples from 10 social groups). Ellipses are the 95% prediction ellipses corrected for sample size (ell95c) calculated from these data for each social group.

Banded mongooses varied greatly in the isotopic composition of their vibrissae, both for δ^13^C (range = − 20.45‰ to − 15.63‰) and δ^15^N (range = 8.05‰ to 14.89‰; Fig. 3). We observed marked variation in isotope values both between social groups (Fig. 3) and between individuals within social groups (Fig. 1a).

**Figure 4:**
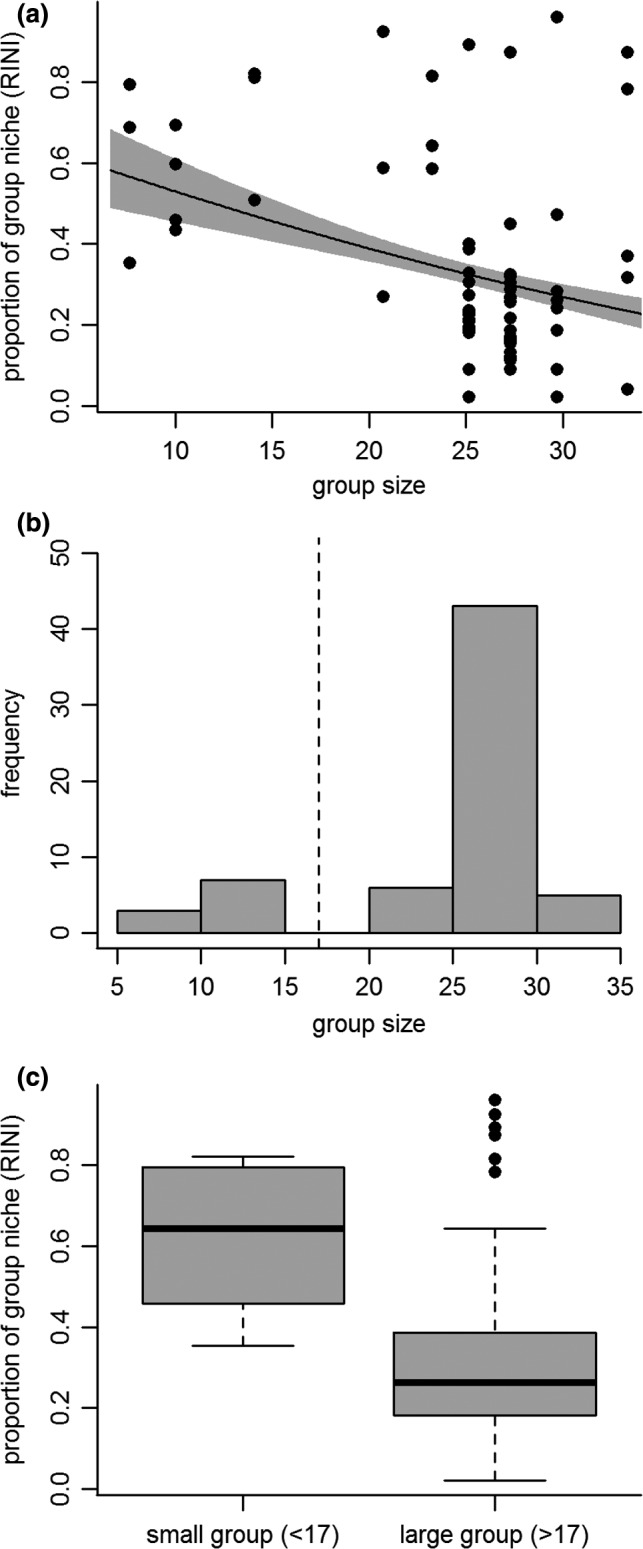
Individual banded mongooses (*Mungos* mungo) in larger groups have smaller foraging niches, measured as a proportion of their group's niche (RINI, see Fig. 1). Panel a shows the raw data (points; 315 samples from 64 individuals) and relationship ± SE (line and shaded area) predicted by our model with group size as a continuous variable with all other variables set at their mean. Group size data were bimodal (panel b) and so we divided our data into individuals from small and large groups (denoted by dotted line at 17 individuals in panel b) and refitted our model with group as a categorical. This also showed that individuals in larger groups occupied smaller foraging niches (panel c). The box‐and‐whisker plot in panel c shows the median (thick horizontal line), interquartile range (boxes) and 1.5 times the interquartile range (whiskers) for the data in small and large groups. The points show data that fall outside of this range.

In support of the ‘niche partitioning’ hypothesis, individuals in larger social groups displayed smaller isotopic niches relative to group isotopic niche (LMM: β ± SE = − 0.010 ± 0.003, χ^2^_1_ = 9.51, *P* = 0.002; Table 2; Fig. 4a). Individuals’ relative niche did not vary with age (LMM: χ^2^_1_ = 1.49, *P* = 0.22) or sex (LMM: χ^2^_1_ = 0.89, *P* = 0.35). The group size data were bimodal (Fig. 4b) and so we refitted our model with group size categorised as small or large (either side of the dotted line at 17 individuals in Fig. 4b). These results were consistent with that of our continuous group size model, showing that individuals in large groups had smaller isotopic niches (LMM: β ± SE = −0.17 ± 0.064, χ^2^_1_ = 7.51, *P* = 0.006; Table 2, Fig. 4c). Group size did not affect a group's overall isotopic niche size fitted either as a continuous variable (F_1,9_ = 0.059, *P* = 0.82) or as a categorical variable (F_1,9_ = 0.0029, *P* = 0.96; Table 2).

**Table 2:** Linear mixed effect models predicting relative individual niche index (RINI; square‐root transformed) and social group isotopic niche size (ell95c) in banded mongooses

	Response	Effect	Estimate	SE	χ^2^	*P*	Conditional *r* ^2^
Individual‐level	RINI (sqrt)	Intercept	0.94	0.10			0.33
		Age	0.017	0.014	1.59	0.22	
		Sex (male)	0.042	0.046	0.89	0.35	
		**Group size**	−0.010	0.003	9.51	**0.002**	
		**Proportion of group**	−0.40	0.10	12.64	**0.0004**	
	RINI (sqrt)	Intercept	0.82	0.08			0.31
		Age	0.015	0.014	1.13	0.29	
		Sex (male)	0.058	0.046	1.71	0.19	
		**Group size (large)**	−0.17	0.064	7.51	**0.006**	
		**Proportion of group**	−0.37	0.11	10.80	**0.001**	
							
Group‐level	Group ell95c	Intercept	2.78	2.64			0.11
		Group size	−0.011	0.046	0.059	0.82	
		Sampling time	0.0046	0.0047	0.95	0.36	
	Group ell95c	Intercept	3.12	2.97			0.11
		Group size (large)	−0.046	0.86	0.0029	0.96	
		Sampling time	0.0038	0.0043	0.78	0.40	

RINI was calculated as the proportion of the group's niche that the individual niche occupied (see Fig. 1). Social group niche size was calculated using small sample size corrected 95% prediction ellipses (ell95c; Fig. 2). The group size data were bimodal (Fig. 3b) and so models were fitted using a continuous and a categorical group size variable. Significant effects are shown in bold.

## Discussion

We found that individual banded mongooses in larger groups had smaller relative isotopic niches (RINIs), despite larger groups occupying similar sized niches to smaller groups. This result supports the prediction of the niche partitioning hypothesis; individuals respond to greater intragroup competition by reducing their niche width, and so reducing their degree of overlap with other group members (Pianka 1974; Schoener 1974; Svanbäck & Bolnick 2007). In many group‐living species, individuals frequently forage in close proximity to one another, resulting in high levels of local competition for resources (de Luca & Ginsberg 2001; Jolles *et al*. 2013; Burke *et al*. 2015; Eshchar *et al*. 2016). Therefore, sociality may play an important role in the development of individual foraging specialisation, as this increase in local competition promotes foraging niche partitioning between group members.

Our results may also suggest that the ability to specialise is a prerequisite for group living. Partitioning of resources through individual specialisation is widely recognised as a mechanism that can decrease competition (Pianka 1974, 1981; Schoener 1974; Correa & Winemiller 2014). A significant cost of living in groups is high local competition between individuals (Krause & Ruxton 2002), therefore niche partitioning of foraging resources may serve to reduce conflict between group members, allowing for their stable coexistence. Without the ability to specialise and reduce niche overlap, the costs of competition associated with group living may be too high, which may explain why we observe foraging specialisation in group‐living species such as banded mongooses.

Our results do not support the optimal foraging hypothesis, which predicts that with increased competition, individuals should add new prey items to their diet, increasing their individual niche (Stephens & Krebs 1986). Recent theoretical models have shown that increased competition can lead to higher levels of foraging specialisation where individuals differ in how they add prey items to their diet (Svanbäck & Bolnick 2005). Where individuals have the same preferences for additional food items, but differ in their propensity to add these items to their diet, the increase in specialisation is expected to be relatively moderate (‘shared preference’ model). Where individuals differ in their preference of additional food items, specialisation is expected to increase with competition more sharply as individuals will add different prey items to their diet (‘competitive refuge’ model) (Svanbäck & Bolnick 2005). (In both cases, this trend reverses at the highest levels of competition due to all prey items becoming depleted.) The prediction that increased competition should lead to greater individual foraging specialisation is supported by empirical studies (e.g. Svanbäck & Bolnick 2007; Tinker *et al*. 2008; reviewed in Araújo *et al*. 2011), suggesting that individuals do differ in how they add prey items to their diet. Identifying rank‐preferences for prey items in group‐living species, and whether they are consistent (‘shared preference’) or variable (‘competitive refuge’) across individuals, will improve our understanding of how intragroup competition influences individual foraging specialisation in animal societies. More generally, this resonates with the increasingly established understanding of the importance of individual differences in ecology and evolution (Dall *et al*. 2012), including in banded mongooses (Sanderson *et al*. 2015).

Although our study suggests that intragroup competition is a driving force in the development of individual specialisation in banded mongooses, the mechanism behind what determines an individual's position in niche space is unclear. Social learning is a widely proposed mechanism behind the development of individual foraging niche (Thornton & Malapert 2009; Tinker *et al*. 2009; Slagsvold & Wiebe 2011; Rossman *et al*. 2015). In both group‐living and non‐social species, individuals learn foraging techniques from their parents (Galef & Laland 2005; Slagsvold & Wiebe 2011; Thornton & Clutton‐Brock 2011; Rossman *et al*. 2015). However, living in groups provides further opportunity to learn from other group members (Krause & Ruxton 2002; Müller & Cant 2010; Farine *et al*. 2015). Whether social learning leads to more or less individual foraging specialisation is expected to depend on how many demonstrators an individual learns from (Cavalli‐Sforza & Feldman 1981). Where individuals each learn from a different specific demonstrator (‘one‐to‐one’ learning) this is expected to lead to greater behavioural heterogeneity and so greater specialisation (e.g. Rossman *et al*. 2015). Social learning may also promote specialisation where exploitation of each prey item requires the learning of a different complex skill and individuals are constrained in their ability to retain multiple skills (Estes *et al*. 2003; Tinker *et al*. 2009). In contrast, where an individual learns from many demonstrators (‘many‐to‐one’ learning) this is expected to lead to behavioural homogeneity and less specialisation (e.g. Hopper *et al*. 2011). Banded mongoose pups form close relationships with adult group members known as escorts, most often not their parents (Vitikainen *et al*. 2017), who care for and feed them until independence (Cant *et al*. 2013, 2016). Past studies have demonstrated that these care‐givers transfer foraging techniques to pups, and that pups retain these preferences after independence (Müller & Cant 2010). That is, they exhibit one‐to‐one social learning (Cavalli‐Sforza & Feldman 1981) which may act alongside the effects of intragroup competition shown in this study to promote individual foraging specialisation. The effect of competition on individual foraging specialisation in social groups may, therefore, depend on the mode of the social learning present within the group.

Differences in individual niche size were not attributable to the age or sex of the individual, suggesting that banded mongooses display true individual foraging specialisation within social groups (Bolnick *et al*. 2003). It is worth noting that individuals can differ in their isotope values due to variation in physiological state. However, the variation between individuals observed in this study (up to Δ6.61‰ for δ^15^N) is much larger than what we would expect from differences in stress‐levels alone (~ Δ0.5–2.0‰, Hobson *et al*. 1993; Δ1.68‰, Cherel *et al*. 2005).

All groups had a similar niche size, suggesting that larger groups did not have access to a greater range of food resources. However, values of both δ^15^N and δ^13^C isotopes did vary between social groups (Fig. 3). That is, group niches varied in location but not size. Banded mongooses occupy distinct territories which they aggressively defend (Thompson *et al*. 2017). Past studies have found that isotope values can vary with geographic location within a study area (Robertson *et al*. 2014; Rossman *et al*. 2015). We suspect, therefore, that territoriality constrains the size of a banded mongoose group's niche and the geographic location of the territory determines the location of this niche with isotopic space. Within banded mongoose groups, however, there are no discernible differences in individual space use as the group travels as a cohesive unit. Instead, our findings show that individual variation in foraging niche within groups is driven by foraging competition. Understanding how the social environment impacts specialisation has important ecological implications. For example, if increased individual differences in foraging preference reduces conflict between group members, then individual specialisation may maintain stable societies and play an important role in the evolution of social systems (Barta 2016).

Our study provides evidence that intragroup competition can lead to greater between‐individual variation in group‐living species; a pattern consistent with competition theory. Individuals in larger groups occupied smaller isotopic niches despite all groups having similar overall niche sizes. This suggests that group‐living species may reduce conflict between group members through niche partitioning with implications for our understanding of the evolution of social behaviour and individual specialisation.

## Authorship

MC, HM, RI and CS designed research; CS performed research; CS and HM undertook stable isotope sample preparation; AJ and RI developed individual foraging metrics; CS and HM analysed data; CS, MC and HM wrote the manuscript; all authors contributed to the final draft.

## Data Accessibility Statement

The data supporting this study has been deposited in Figshare (https://doi.org/10.6084/m9.figshare.5863416).
